# Relationship of BMI to the incidence of hypertension: a 4 years’ cohort study among children in Guangzhou, 2007–2011

**DOI:** 10.1186/s12889-015-1997-6

**Published:** 2015-08-14

**Authors:** Jiao Wang, Yanna Zhu, Jin Jing, Yajun Chen, Jincheng Mai, Stephen H.S. Wong, John O’Reilly, Lu Ma

**Affiliations:** Department of Maternal and Child Health, School of Public Health, Sun Yat-Sen University, 74 Zhongshan Road 2, Guangzhou, 510080 China; Guangzhou Health Care Clinics of Middle and Primary Schools, Guangzhou, China; Department of Sports Science and Physical Education, Chinese University of Hong Kong, Shatin, Hong Kong

**Keywords:** Pediatric, Hypertension, Obesity

## Abstract

**Background:**

In China, there has been a dramatic increase in overweight and obesity among children and adolescents in recent decades. However, little longitudinal studies reported BMI in relation to the risk for hypertension among children in China. We examined the longitudinal relations between BMI and hypertension in Chinese schoolchildren via a retrospective cohort study.

**Methods:**

The cohort study was carried out in 7203 children (3821 boys and 3382 girls) in Guangzhou aged 6–8 years, with a continuous 4 years of follow-up. The participants, evaluated by body mass index (BMI), were categorized as thinness, normal weight, overweight, and obesity groups. The age and gender-specific BMI cutoffs newly developed by the Working Group on Obesity in China (WGOC) were used to define overweight and obesity. The thinness was defined by the international age- and gender-specific cut-off points for BMI for thinness grade 1. Hypertension was defined by using percentiles of systolic and diastolic values on the basis of height percentile, age, and gender. The Cox proportional hazards model was used to estimate the single or joint effect of BMI on the risk of hypertension. This study was approved by The Ethical Committee of School of Public Health, Sun Yat-sen University.

**Results:**

During a follow-up of 4 years, a shocking high cumulative incidence of hypertension was found in Chinese overweight (50.1 %) and obesity (70 %) schoolchildren. The incidence of children hypertension were markedly higher among overweight and obesity group than normal weight and thinness group (24.3 %, 18.5 % vs 11.1 %, 7.4 %). Compared with the children in the normal weight group, the adjusted HRs and 95 % CIs of developing hypertension in thinness, overweight, and obesity group were 0.972 (0.851, 1.110), 1.313 (1.179, 1.461), and 1.816 (1.634, 2.081), respectively. Additionally, the protective effect of thinness on hypertension was observed in boys 0.808 (0.666, 0.981), but not in girls 1.158 (0.966, 1.389).

**Conclusions:**

The 4-year longitudinal study indicated that the overweight and obesity can predict the higher risk of hypertension in Chinese children, whereas, the thinness predict the lower risk of hypertension only in boys.

## Background

Overweight and obesity were now considered as serious health problems, with an increasing prevalence worldwide. In China, there has been a dramatic increase in overweight and obesity among children and adolescents worldwide in recent decades [1]. The prevalence for overweight in 2002 was 22.8 % and for obesity 7.1 %, has increased by 40.7 % and 97.2 %, respectively, since 1992 [2]. However, the prevalence of stunting and thinness among Chinese children and adolescents aged 5–19 years has reached 13.8 %, and 7.4 % in 2007, respectively [3]. This novel and complex problem challenges governments and health organizations to tackle opposite ends of the malnutrition spectrum. The dual burden may manifest within a community, household, or individual, but these different levels have not been addressed collectively [4].

Increasing evidence suggests that this epidemic of childhood obesity was causing premature onset of hypertension, resulting in increased risk for adult coronary heart diseases [5, 6]. The landmark Global Burden of Disease Study showed that the hypertension now tops the list of risk factors for death and disability worldwide [7]. The prevalence of hypertension increased dramatically from 1991 to 2004, with average relative increase of 8.13 % in Chinese children and adolescents [8]. Importantly, more and more evidence showed hypertensive children are more likely to develop hypertension in adulthood [9, 10].

Body mass index (BMI) is often the measure of thinness and obesity used in some literatures even though it is a ratio of weight to height. Although some cohort studies have shown that the higher the BMI, the greater the likelihood of developing hypertension in adults [11–17], few longitudinal studies evaluated BMI across the range of thinness and obesity as a primary risk factor. In addition, there were few studies with samples which were powerful enough and a dropout rate low enough in their follow up. Guangzhou is a big coast city located on the south of China. In this study, we observed the trend of childhood obesity and hypertension development, and thus examined the longitudinal relations between BMI and hypertension in Chinese primary schoolchildren via a retrospective cohort study.

## Methods and procedures

### Design and participants

The retrospective cohort study was conducted in 54 primary schools from 7 districts in Guangzhou city of China from January 2007 until December 2011. The anthropometric, pathological treatment, and prognostic data of 8118 subjects aged 6 to 8 years children were collected retrospectively from Guangzhou Health Care Clinics of Middle and Primary Schools, excluding subjects with a diagnosis of hypertension (*n* = 495) [18], use of antihypertensive drugs (*n* = 6), a history of heart failure (*n* = 4), and incomplete data on any other variable required in this study (*n* = 32). Participants of the cohort underwent routine examinations approximately every year. A total of 7203 children were recruited in final study cohort in 2011 year. Written informed consent form was obtained from adolescents and their parents. This study was approved by the University Ethical Committee.

### Measures and definitions

Trained study staff measured each participant’s height, weight, and BP by using standardized protocols. Standing height with shoes removed was measured with a measuring tape to the nearest millimeter. Body weight was measured to the nearest 0.1 kg on calibrated digital scales. BMI was computed by dividing weight (kg) by height squared (m^2^). The age and gender-specific BMI cutoffs newly developed by the Working Group on Obesity in China (WGOC) were used to define overweight and obesity [19]. Overweight is defined as a BMI at or above the 85th percentile and lower than the 95th percentile for children and adolescents of the same age and sex. And obesity is defined as BMI greater than the 95th percentile. The thinness was defined by the international age- and gender-specific cut-off points for BMI, for thinness grade 1 [20].

Blood pressure (BP) was measured by trained physicians using a standard mercury sphygmomanometer at the right arm with students in the seated position after at least 5 min of rest. The cuff size was based on the length and circumference of the upper arm and was chosen to be as large as possible without having the elbow skin crease obstruct the stethoscope [21]. Blood pressure values were approximated to the nearest 2 mmHg. In order to avoid ‘white-coat’ effect on blood pressure and make the children comfortable in a relaxed environment, measurements were taken in the classroom in the presence of their classmates and teachers and doctors wore casual clothes. Systolic blood pressure (SBP) was defined by the first Korotkoff sound (appearance of sounds), and diastolic blood pressure (DBP) was defined by the fifth Korotkoff sound (disappearance of sounds). According to National High Blood Pressure Education Program in USA, hypertension was defined by using percentiles of systolic and diastolic values on the basis of height percentile, age, and gender [18]. In this study, hypertension is defined as average SBP and/or DBP that is ≥ 95th percentile for gender, age, and height on 3 occasions. Three measurements were taken at 1-min intervals. The average of the second and third measurements was taken as the pressure of record.

### Statistical analysis

The continuous variables, including age, weight, height, BMI, and blood pressure, were described by means and standard deviations. Categorical variables were displayed by count and percentage. Chi square test was performed to compare the difference of categorical variables between boys and girls. Furthermore, the Cox proportional hazards model was used to estimate the single or joint effect of nutritional status on the risk of hypertension incidence. The reference category in the Cox proportional hazards model was normal weight BMI group. In this model, the hazards ratios of the total population were adjusted for age, gender and baseline blood pressure, and those of the boys or girls were adjusted for age and baseline blood pressure, and the age was adjusted by 1-year categorical variable. All data were analyzed by using SPSS 13.0 (SPSS Inc., Chicago, Illinois, USA). A *p*-value of 0.05 was accepted as indicating statistical significance.

## Results

The demographic characteristics of the participants at baseline are shown in Table 1. The boys and girls, with the same average age of 6.6 ± 0.6 years, accounted for 53.0 % (*n* = 3821) and 47.0 % (*n* = 3382) of the total percentage, respectively (*P* > 0.05). Compared with girls, boys had higher weight, height, BMI, SBP, and DBP (all *P* < 0.05). In addition, the prevalence of thinness, normal-weight, overweight and obesity among boys was 18.5 %, 64.5 %, 8.6 %, and 8.4 %, while it was 18.8 %, 70.1 %, 6.1 % and 5.0 % among girls, respectively (*P* < 0.05).

**Table 1:** Baseline characteristics of studied children in Guangzhou^a^

Characteristics	Overall	Boys	Girls	P Value*
N (%)	7203	3821(53.0)	3382(47.0)	0.31
Age (year)	6.6 ± 0.6	6.6 ± 0.6	6.6 ± 0.6	0.13
Weight (kg)	25.2 ± 4.8	27.2 ± 5.1	23.7 ± 4.2	< 0.001
Height (m)	1.25 ± 0.06	1.27 ± 0.059	1.22 ± 0.059	< 0.001
BMI (kg/m^2^)	15.4 ± 2.1	15.8 ± 2.2	14.9 ± 1.9	< 0.001
Thinness (%)	18.6	18.5	18.8	< 0.001
Normal weight (%)	67.4	64.5	70.1	< 0.001
Overweight (%)	7.7	8.6	6.1	< 0.001
Obesity (%)	6.3	8.4	5.0	< 0.001
SBP(mm/Hg)	96.9 ± 6.1	98.5 ± 6.5	94.1 ± 6.3	< 0.001
DBP(mm/Hg)	56.1 ± 7.3	56.6 ± 7.3	54.5 ± 7.0	< 0.001

a. Data in this table, unless otherwise specified, are given as means and standard deviations

P Value* from independent samples t-test among both genders

**Fig. 1:**
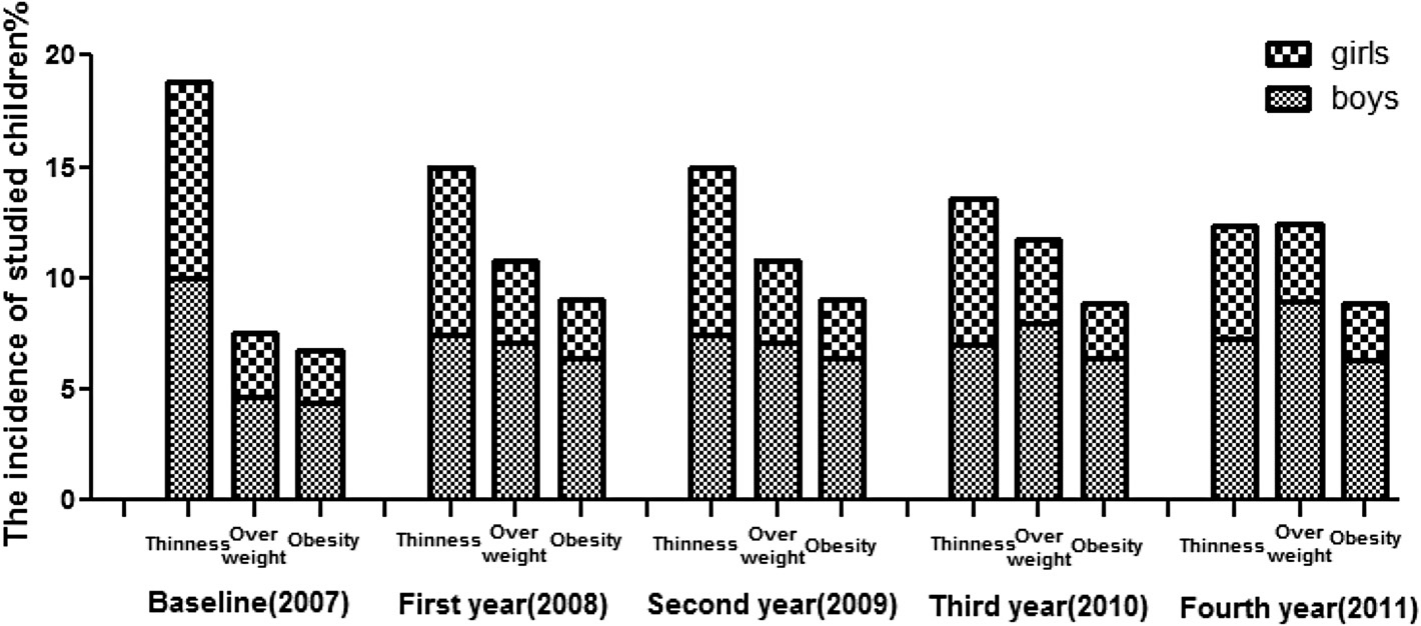
The different BMI groups of studied children during the follow-up period^a,b^. **a** Data in this table figure were given as percentages, displaying the incidence of studied children by follow-up year and BMI groups. **b** The incidence of boys and girls were calculated among overall population

Figure 1 shows the percentage of boys and girls students with thinness, overweight and obesity during the 4 follow-up years. The overall incidence of thinness, overweight, and obesity among students was 18.6 %, 7.7 % and 6.3 % at the first year in 2007, while it was 12.3 %, 12.4 % and 8.3 % at the final year in 2011. There was a significant decrease for incidence of thinness in both boys and girls that corresponded with increased results during the follow-up years. In contrast, the incidence of overweight and obesity increased significantly with the follow-up years. The boys had a higher incidence of overweight (*P* < 0.05) and obesity (*P* < 0.05) than girls in most of the follow-up years.

The hypertension incidences of children according to nutritional status during the follow-up period are displayed in Table 2. Among 7203 participants, 993 (13.7 %), 1015 (16.3 %), 462 (8.8 %), and 303 (6.4 %) students developed hypertension during the 4 follow-up years, respectively. The incidence of hypertension was markedly higher among obese children in overall population (24.3 %) in the first follow-up year, compared with children in the overweight group (18.5 %), in the normal weight group (11.1 %) and in the thinness group (7.4 %). Meanwhile, the incidences of hypertension increased significantly with the degree of BMI groups. These findings were generally consistent throughout the 4 follow-up years both in boys and girls. Furthermore, mostly in the overweight and obesity groups whether in boys or girls, the incidences of hypertension increased at the second follow-up year with an average age of 8.6 years in 2009 compared with the baseline, but decreased in the following years of 2010 and 2011.

**Table 2:** The hypertension incidence of studied children according to BMI groups during the follow-up period

	Overall				Boys				Girls			
BMI groups	(2008)	(2009)	(2010)	(2011)	(2008)	(2009)	(2010)	(2011)	(2008)	(2009)	(2010)	(2011)
	First	Second	Third	Fourth	First	Second	Third	Fourth	First	Second	Third	Fourth
	year	year	year	year	year	year	year	year	year	year	year	year
	(n = 7203)	(n = 6210)	(n = 5195)	(n = 4733)	(n = 3821)	(n = 3307)	(n = 2758)	(n = 2512)	(n = 3382)	(n = 2903)	(n = 2437)	(n = 2221)
Thinness, n (%)	95(7.4)	92(8.2)	46(5.0)	23(3.0)	46(7.1)	39(7.0)	24(4.9)	11(2.3)	49(7.8)	53(9.3)	22(5.1)	12(4.3)
Normal, n (%)	601(11.1)	522(11.1)	278(6.6)	177(4.4)	278(10.1)	233(9.9)	131(6.3)	68(3.7)	323(12.1)	289(12.4)	147(6.9)	109(5.1)
Overweight, n (%)	133(18.5)	180(24.3)	81(12.7)	51(8.3)	72(16.3)	114(22.8)	51(11.1)	36(7.4)	61(22.0)	66(27.4)	30(16.9)	15(9.3)
Obesity, n (%)	164(24.3)	221(39.0)	57(17.4)	52(19.1)	118(25.3)	163(40.5)	40(16.9)	29(15.0)	46(21.9)	58(35.4)	17(18.9)	23(29.1)
Total, n (%)	993(13.7)	1015(16.3)	462(8.8)	303(6.4)	514(13.4)	549(16.6)	246(8.9)	144(5.7)	479(14.1)	466(16.0)	216(8.8)	159(7.2)

Among the 7203 school-aged children (3821 boys and 3382 girls), 2773 children (38.5 %) developed hypertension (1453 boys and 1320 girls) totally during the follow-up years. Table 3 shows sex-stratified cumulative incidence of hypertension according to the nutrition status. The cumulative incidence of hypertension increased significantly with the degree of nutritional status in overall population and both genders groups. Additionally, the girls had a higher cumulative incidence of hypertension than boys in thinness and overweight groups (*P* < 0.05).

**Table 3:** The cumulative incidence of hypertension in studied children at the end of follow-up

	Overall		Boys		Girls		
BMI groups	n	%	n	%	n	%	P Value*
Thinness	256	22.3	120	19.9	136	24.9	< 0.001
Normal	1578	29.4	710	27.1	868	31.6	0.13
Overweight	445	50.1	273	45.8	172	59.1	< 0.001
Obesity	494	70.0	350	69.4	144	70.8	0.22
Total	2773	38.5	1453	38.0	1320	39.0	0.38

P-values* from Chi-square tests for categorical variables between boys and girls

**Fig. 2:**
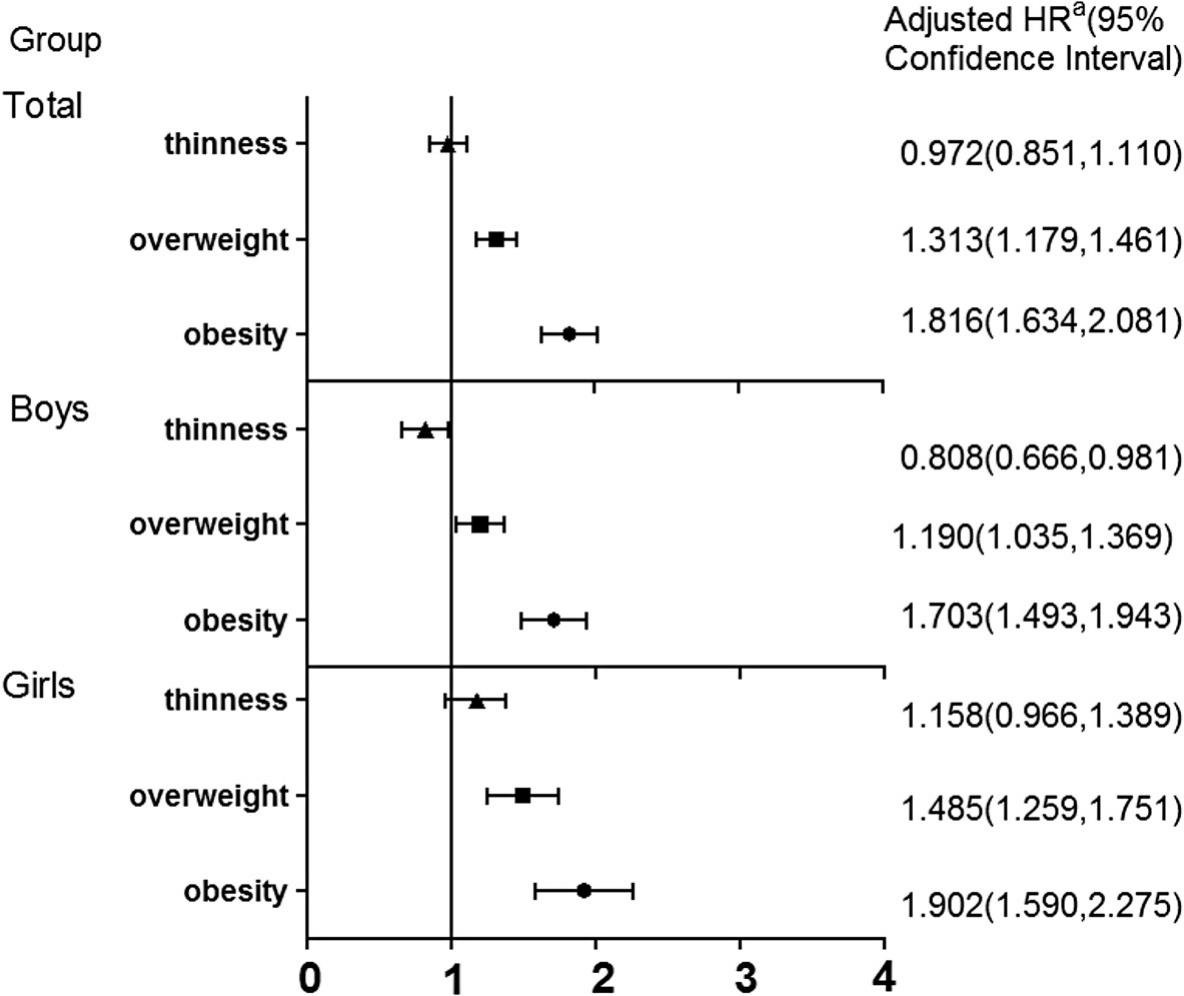
Hazards ratios of hypertension according to different BMI groups^b^. **a** Reference category: normal weight BMI. **b** The Hazards ratios of the total population were adjusted for age, gender and baseline blood pressure, and those of the boys or girls were adjusted for ages and baseline blood pressure

Cox proportional hazards model results were shown in Fig. 2. After adjustment for age gender and baseline blood pressure, in comparison with normal weight students, the Hazards ratios (HRs) of hypertension was 1.816 (95 % CI 1.634–2.081, *P* < 0.05) and 1.313 (95 % CI 1.179–1.461, *P* < 0.05) in obesity and overweight students, respectively, which indicated an increased risk of developing hypertension in the two status. In addition, compared to normal weight students, there was also a slight association between thinness and hypertension with a decreased risk of developing hypertension in boys (adjusted HR = 0.808, 95 % CI = 0.666–0.981, *P* < 0.05).

## Discussion

A shockingly high cumulative incidence of hypertension was observed in Chinese overweight (50.1 %) and obesity (70 %) schoolchildren during a follow-up of 4 years from 2007 to 2011. The present study showed that the risk of hypertension was closely related to BMI in Chinese primary schoolchildren. Generally, the hypertension incidences in primary schoolchildren were markedly higher among overweight and obesity group than normal weight and thinness group. Meanwhile, the Cox proportional hazards model indicated that the overweight and obesity status can predict a higher risk of hypertension in Chinese primary schoolchildren, while thinness predicted a lower risk of hypertension in boys.

In this cohort study, we observed that the cumulative incidence of hypertension in normal weight, overweight and obesity group has reached 29.4 %, 50.1 % and 70.0 % in overall population in our study, respectively. Although several cohort studies have reported an association between obesity and risk of hypertension, less information was provided on the cumulative incidences of hypertension in different BMI group status’ of children. Almost 20 % of the children with normal BP values showed elevated BP values in one 6-year BP tracking study [22], which is lower than the cumulative incidence of hypertension in our findings. Another study [23] reported the cumulative 8-year hypertension incidence in obese individuals was lower than what have been reported in this study. These contradictory results may be explained by differences in the definitions of obesity and hypertension and differences in age, sex, ethnicity, and size of the study samples. In particular, the criteria for defining children’s hypertension is different among various studies. Because of the lack of the widely recognized definition of children’s hypertension in China, we used the international normative definition in this study, according to the latest studies of the National High Blood Pressure Education Program (NHBPEP) Working Group on High Blood Pressure in Children and Adolescent [18], which was widely used in US-based or European-based studies. The differences in incidences of hypertension could partially be explained by this reason.

A positive association between BMI and BP was found in our study. Although there were a number of observational studies which have examined the relationship between obesity and hypertension, limited longitudinal studies were available, especially in Chinese Children. Among large-scale population of children living in Guangzhou who were examined in this study, we observed a strong and positive association between obesity and hypertension. And the incidences of hypertension were higher in overweight and obese children, when compared with children of another status throughout both genders. Our findings are consistent with previous studies in Caucasian children, such as the Harvard growth study in 1992 [24], Mexico City schools study in 2009 [25], and Indianapolis in 2011 [26]. These results suggested that increased overweight and obesity will undoubtedly push up the prevalence of chronic diseases like hypertension.

Another striking finding of this study is that the lowest incidence of hypertension was observed among thin students compared with other groups throughout all follow-up years, and the thinness status predicted a lower risk of hypertension in boys. However, one study conducted in Hainan province in 2012 showed that compared to normal weight students, no significant association between thinness and hypertension was observed [27]. Another cross-sectional study conducted in India showed that thin children, identified by any reference, have a lower risk of associated cardio-metabolic aberrations [28]. The inconsistency across these studies might be partially attributed to their differences in research design and study population. Furthermore, the causal relationship between thinness and elevated blood pressure cannot be concluded in the cross-sectional study. Rather, a longitudinal study design could help to solve this question. The gender differences in the association between thinness and hypertension maybe because of the markedly reduced body fat percentage (BF %) in their lean girls, but it was still hard to exclude the prevalence of thinness. So the gender differences in the association between thinness and hypertension merit exploration in future.

The mechanism by which BMI decreased has caused the remission of hypertension is to remain unclear. Fat mass might cause an elevated blood pressure by raising cardiac output with relatively increased peripheral vascular resistance [29, 30]. However, weight loss may decrease cardiac output and peripheral vascular resistance due to a decreasing fat mass. Additionally, weight loss may also impact the remission of hypertension through its role in decreasing sympathetic nervous system activity, insulin resistance and hyperinsulinemia, sodium retention and enhanced vascular reactivity [31]. Therefore, there were the lowest incidences of hypertension among thinness students.

In the current study, the overall prevalence of children hypertension was 6.1 % in 2007, which was relatively lower than those reported from other areas of China, i.e., 13.63 % in Hangzhou [32], 10.26 % in Shenzhen [33]. Furthermore, the variability in serial BP measurements in children also can be found in our study. The incidences of hypertension increase at the second follow-up year with an average age of 8.6 years in 2009, but decreased during the following two years, mostly in the overweight and obesity groups. The hypertension incidence line in the last 2 years began to diverge significantly from the line before, implying that puberty could be an increased risk for hypertension.

Compared to inland cities in China, the prevalence rates of overweight and obesity (7.7 % and 6.3 %, respectively) in Guangzhou are relatively high, i.e., the obesity prevalence among school students was 4.11 % in Xi’an [34]. But they are still lower when compared to other big cities in China and developed countries. In China, the schoolchildren overweight and obesity prevalence was 11.27 %, and 13.53 %, respectively in Shanghai in 2009 [35], and 12.5 % and 15.7 % in Tianjin in 2011 [36]. For other countries, the overweight prevalence is 30.8 % in Spanish schoolchildren, 31 % in Greece, 36 % in Italy, 19 % in France, 16 % in Germany, 15 % in Denmark, and 25.6 % in USA [37]. Different cultures and racial characteristics, and widely disparate eating habits and lifestyles including diet, salt intake and levels of physical activity which would affect incidence of overweight and obesity should been taken into account. Now there are evidences that childhood obesity can be prevented or modified at a population level [38].

The incidence of childhood overweight and obesity has increased over time, while the incidence of thinness has decreased in the present study. Similar trends were found in some other studies. A 6-year longitudinal cohort of Australian primary schoolchildren showed a trend for obesity to increase in the mid socioeconomic status group over the 6 years from 2007 to 2012 [39]. This phenomenon could be explained by the body fat percentage (%BF) curves in children and adolescents [40]. In general, %BF for boys increased throughout middle to late childhood and peaked at approximately age 11 years and girls displayed a similar pattern of age-related changes in %BF compared to boys through about age 9 years.

The present study has several limitations. One limitation was the lack of a measure of central adiposity, such as waist circumference. Meanwhile, some potential confounders like dietary intake of salt, fat, sugar, physical activity and parental socioeconomic status were not investigated or controlled except for age and sex because of the lack of details in this study. The independent association between weight statutes and hypertension should be shown in multiple analysis taking these confounders especially physical activity, genetic susceptibility and salt intake into account. These information will be included in the following follow up study conducted in high school students.

## Conclusion

In summary, the 4-year longitudinal study indicates that the overweight and obesity can predict a higher risk of hypertension in Chinese children, whereas thinness slightly predicts a lower risk of hypertension in boys. Although the BMI status are varied in different areas, they can have a significant impact on incidence of hypertension among school-aged children.

## Abbreviations

BP: Blood pressure
HRs: Hazards ratios
CIs: Confidence intervals
BMI: Body mass index
SBP: Systolic blood pressure
DBP: Diastolic blood pressure
%BF: Body fat percentile
